# Targeting G2/M checkpoint vulnerability in homologous recombination-proficient tumors: lessons from ovarian cancer models

**DOI:** 10.1080/15384101.2026.2751743

**Published:** 2026-10-27

**Authors:** Qian Xi, Akiko Kunita, Saeko Nagai, Anh Quynh Duong, Masanori Kawakami, Katsutoshi Oda

**Affiliations:** a Division of Integrative Genomics, Graduate School of Medicine, The University of Tokyo, Tokyo, Japan; b Department of Gynecological Oncology, Sun Yat-sen University Cancer Center, Guangzhou, China; c Next-Generation Precision Medicine Development Laboratory, Graduate School of Medicine, The University of Tokyo, Tokyo, Japan; d Department of Pathology, Graduate School of Medicine, The University of Tokyo, Tokyo, Japan; e Department of Obstetrics and Gynecology, Graduate School of Medicine, The University of Tokyo, Tokyo, Japan; f Department of Respiratory Medicine, Graduate School of Medicine, The University of Tokyo, Tokyo, Japan

**Keywords:** G2/M checkpoint, PLK1 inhibitor, WEE1 inhibitor, homologous recombination, ovarian cancer, replication stress

## Abstract

Cell cycle dysregulation is a hallmark of cancer; however, tumors retaining homologous recombination (HR)-proficiency remain a major challenge. Although PARP inhibitors effectively target HR-deficient cancers, targeted therapies for *BRCA1*/*2*-wild-type, HR-proficient tumors are limited. High-grade serous ovarian cancer, approximately half of which is HR-proficient, illustrates that HR proficiency, together with replication stress and checkpoint dependence, may represent both a mechanism of therapeutic resistance and a druggable dependency on checkpoint-coupled DNA repair. This review synthesizes evidence that targeting either PLK1 or WEE1 independently exposes this vulnerability. PLK1 and WEE1 inhibition engage distinct mechanisms: PLK1 inhibition disrupts spindle assembly and HR competence, whereas WEE1 inhibition amplifies replication stress and forces premature mitotic entry. Nevertheless, both ultimately converge to compromise HR-associated repair, promote unresolved DNA damage, and trigger lethal mitotic catastrophe. Finally, we discuss biomarker-unselected trial failures, functional and replication stress-based patient stratification, and rational combinations with DNA-damaging agents and immunotherapy.

## 1. Introduction

### 1.1. Cell cycle checkpoints as therapeutic targets in cancer

Accurate cell cycle control is essential for maintaining genomic integrity, and its disruption is a defining feature of tumorigenesis [1,2]. Loss of genomic integrity contributes to cancer development through dysregulation of the DNA damage response and replication stress pathways, providing a rationale for therapeutically targeting these processes [3,4]. The clinical success of cyclin-dependent kinase 4/6 (CDK4/6) inhibitors (palbociclib, ribociclib, and abemaciclib) in hormone receptor-positive, HER2-negative breast cancer has been validated as a therapeutic strategy for solid tumors and provides a precedent for targeting cell cycle regulatory nodes, although the biology and therapeutic liabilities of the G2/M axis are distinct [5–10]. Unlike CDK4/6 inhibition, the clinical application of which is largely determined by lineage and hormone receptor status, G2/M checkpoint inhibition exploits the functional vulnerability arising from checkpoint dependence. Tumor cells rely more heavily on the G2/M checkpoint than normal proliferating tissues, providing a potential therapeutic window for selective checkpoint inhibition. This dependence is particularly evident in cancers with defective G1/S checkpoint control. Accordingly, many solid tumors harbor *TP53* alterations. In high-grade serous ovarian cancer (HGSOC), *TP53* mutations occur in 96% of cases [11,12]. Loss of p53 function compromises the G1/S checkpoint and shifts survival dependence to the G2/M checkpoint. Importantly, this therapeutic vulnerability extends beyond homologous recombination-deficient (HR-deficient) tumors to homologous recombination-proficient (HR-proficient) tumors, which retain functional *BRCA1/2* and intact HR machinery, account for approximately 50% of HGSOC cases, and currently lack effective targeted therapies.

### 1.2. DNA repair pathway switching and therapeutic vulnerability

Double-strand breaks (DSBs) are repaired through cell cycle-regulated pathways with distinct activities and efficiencies across the cell cycle (Figure 1). HR mainly operates in the S and G2 phases using the sister chromatid as a high-fidelity template. Non-homologous end joining (NHEJ) ligates DNA ends without homology and is predominant in G1. Theta-mediated end joining (TMEJ), which relies on DNA polymerase theta (Polθ) and short microhomologies (2–6 bp) [13], becomes particularly important during mitosis when HR and NHEJ are substantially constrained [14,15]. Notably, PLK1 positively regulates TMEJ in mitosis by directly phosphorylating Polθ, which enables its recruitment to mitotic DSBs through binding to the BRCT7-8 domains of TOPBP1 [15].

**Figure 1.**
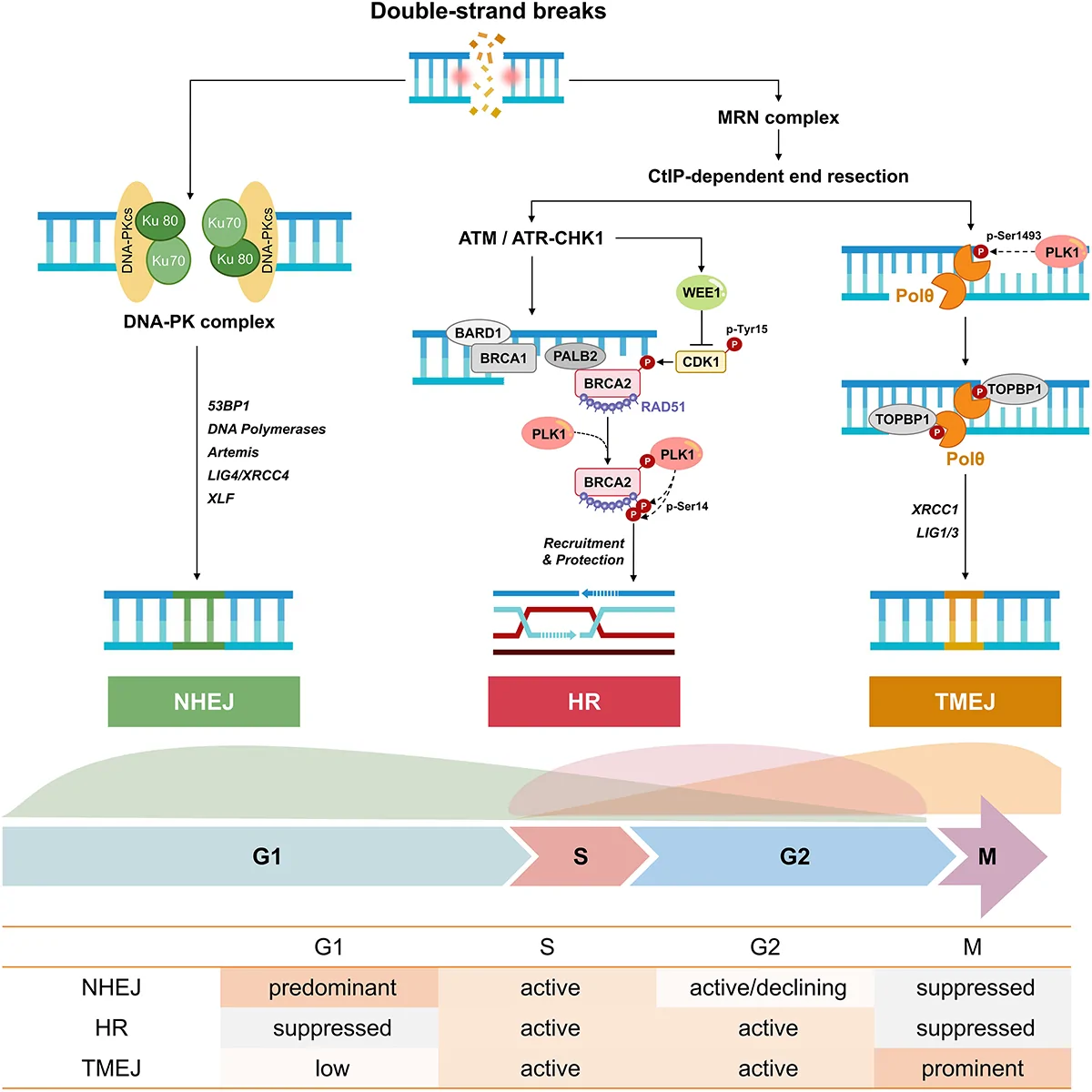
Cell-cycle-dependent DSB repair pathway choice and the roles of PLK1 and WEE1. Double-strand breaks (DSBs) are repaired by three major pathways whose relative usage shifts across the cell cycle. Canonical non-homologous end joining (NHEJ), mediated by the DNA-PK complex (Ku70/Ku80/DNA-PKcs), predominates in G1 and is strongly suppressed in mitosis. Homologous recombination (HR), initiated by MRN/CtIP-dependent end resection and RAD51 loading through the BARD1–BRCA1–PALB2–BRCA2 axis, is active in S/G2 and suppressed in mitosis. Polθ-mediated end joining (TMEJ) operates in S/G2 but becomes prominent in mitosis when NHEJ and HR are constrained. WEE1 preserves the G2/M checkpoint through inhibitory phosphorylation of CDK1 at Tyr15, thereby maintaining CDK activity thresholds that support HR competence in S/G2. PLK1 contributes to HR via a CDK-primed mechanism in which BRCA2 phosphorylation recruits PLK1, which phosphorylates RAD51 at Ser14 using BRCA2 as a molecular scaffold, promoting RAD51 recruitment and HR competence. PLK1 has also been implicated in TMEJ through phosphorylation of Pol θ during mitosis. The lower panel summarizes the relative activity of NHEJ, HR, and TMEJ across cell cycle phases. TMEJ is also discussed in the context of microhomology-mediated end joining (MMEJ).

Both WEE1 and PLK1 are central integrators of this landscape within the G2/M phase, although they act through distinct proximal mechanisms. WEE1 preserves the G2/M checkpoint by inhibitory phosphorylation of cyclin-dependent kinase 1 (CDK1) at Tyr15 and supports HR by restraining CDK activity, thereby maintaining BRCA2-dependent RAD51 loading in the S and G2 [16,17]. PLK1 promotes checkpoint recovery by activating CDC25C and by targeting WEE1 and Claspin for degradation, thereby restoring CDK1 activity. PLK1 also facilitates HR by phosphorylating RAD51 at Ser14 and enables Polθ-dependent TMEJ during mitosis [15,18]. By coordinating checkpoint control with DNA repair pathway selection through different mechanisms, these kinases enable ordered progression throughout the cell cycle. Consequently, the inhibition of either kinase can disrupt the coordination between checkpoint control and DNA repair capacity, creating therapeutic vulnerabilities.

### 1.3. HR proficiency: from resistance mechanism to therapeutic target

The discovery of synthetic lethality between PARP inhibition and *BRCA1/2* deficiency established HR deficiency as a therapeutic target [19]. However, approximately 50% of patients with HGSOC harbor wild-type (WT) *BRCA1/2* and maintain HR proficiency, which has conventionally been viewed solely as a resistance mechanism. We therefore propose a conceptual reframing: HR proficiency may represent a context-dependent therapeutic dependency when checkpoint-associated repair functions are disrupted. However, HR proficiency alone may not fully explain this vulnerability. Co-occurring factors such as Cyclin E1/*CCNE1*-driven replication stress and loss of G1/S checkpoint control are also likely to contribute (Section 2.3). In our recent mechanistic study, either PLK1 or WEE1 inhibition reduced HR-associated repair capacity, promoted DNA damage accumulation, and triggered mitotic catastrophe in *BRCA1/2*-WT/HR-proficient ovarian cancer models, whereas *BRCA1/2*-mutant/HR-deficient cells remained relatively resistant, possibly owing to compensatory DNA-PKcs-dependent NHEJ [20]. Although drawn from a limited set of experimental models, these findings support G2/M checkpoint inhibition as a strategy for exploiting checkpoint-dependent vulnerabilities in HR-proficient tumors.

### 1.4. Scope of this review

This review summarizes the molecular mechanisms linking PLK1 and WEE1 to G2/M checkpoint control and DNA repair pathway selection, examines cancer-selective dependencies and clinical developments, discusses functional biomarker strategies, and outlines rational combinations of approaches. Although ovarian cancer is the primary disease model, these concepts may be applied to other *TP53*-altered HR-proficient solid tumors, pending clinical validation.

## 2. PLK1 and WEE1: G2/M checkpoint kinases at the intersection of mitosis and DNA repair

### 2.1. PLK1: expression and mechanistic roles in mitosis and DNA repair

In normal proliferating cells, PLK1 expression is tightly regulated by the cell cycle, rising in the G2 phase and peaking during mitosis [21]. By contrast, PLK1 is frequently overexpressed in multiple human cancers, including ovarian cancer, breast cancer, glioma, and lung cancer, and its elevated expression correlates with aggressive clinicopathological features and poor clinical outcomes [22,23].

Beyond its expression pattern, PLK1 plays a central role in mitotic entry and progression and is essential for centrosome maturation and spindle assembly [24,25]. PLK1 also regulates DNA repair pathway selection across the cell cycle (Figure 1): it promotes HR through phosphorylation of RAD51, thereby facilitating RAD51 recruitment and loading [18], and as described in Section 1.2, promotes checkpoint recovery through CDC25C activation and activates TMEJ in mitosis by phosphorylating Polθ [15,18]. Collectively, these functions link PLK1-mediated mitotic control with DNA repair competence.

### 2.2. WEE1: expression and mechanistic roles in replication and checkpoint control

WEE1 represents a related but mechanistically distinct checkpoint dependency. Similar to PLK1, WEE1 expression is frequently elevated in ovarian cancer and other malignancies relative to normal tissues [20]. In normal cells, WEE1 inhibits CDK1/2 activity in response to DNA damage or replication stress, thereby preventing premature entry into mitosis. Mechanistically, WEE1 limits CDK2 activity during the S phase, thereby restraining unscheduled origin firing, and inhibits CDK1 at the G2/M transition to maintain checkpoint control [26,27]. In tumor cells, WEE1 becomes a critical stress-buffering dependency, particularly when G1/S checkpoint control is compromised. This dependence is especially pronounced in *TP53*-mutant or Cyclin E1-elevated tumors, which rely disproportionately on G2/M checkpoint control [28–30]. In post-chemotherapy ovarian cancer, high WEE1 expression was predictive of poor survival [31]. In HGSOC, *CCNE1* amplification or Cyclin E1 overexpression is particularly relevant because it enhances CDK2-driven replication stress and is frequently associated with HR-proficient disease [20,29,30]. WEE1 sustains BRCA2-dependent RAD51 loading by maintaining CDK activity at the levels required for HR protein assembly in the S and G2 phases [16,17]. Recent work in fission yeast revealed that Wee1 contributes to kinetochore–microtubule stability independently of Cdc2 (the fission yeast CDK1 ortholog) Tyr15 phosphorylation. Wee1-deficient fission yeast cells display defective kinetochore–microtubule attachment and impaired chromosomal biorientation, leading to constitutive spindle assembly checkpoint activation [32]. These findings raise the possibility that WEE1 inhibition in mammalian cells may compromise spindle integrity through mechanisms beyond checkpoint abrogation; however, the relevance of this yeast-specific observation in mammalian cancer cells remains to be established, and this mechanism has not yet been directly tested in ovarian cancer models.

### 2.3. Convergence and divergence between PLK1 and WEE1

Although PLK1 and WEE1 act through distinct upstream mechanisms, inhibition of either kinase can produce overlapping downstream phenotypes in the HR-proficient models examined, including impaired HR-associated repair, accumulation of DNA damage, and mitotic catastrophe (Table 1). This convergence should not be considered evidence of a shared or interchangeable molecular mechanism; rather, PLK1 or WEE1 inhibition disrupts distinct upstream nodes, leading to overlapping downstream phenotypes. This distinction is particularly relevant for biomarker development: Cyclin E1/*CCNE1* status has a direct mechanistic link to WEE1 inhibitor sensitivity through CDK2-driven replication stress [29,30], whereas the molecular basis linking Cyclin E1 to PLK1 inhibitor sensitivity remains unclear (Section 4.2).

**Table 1.** Comparison of PLK1 and WEE1 inhibition.

Feature	PLK1 inhibition	WEE1 inhibition
Principal cell-cycle functions	Centrosome maturation, spindle assembly, chromosome segregation, and mitotic progression	Restraint of CDK1/2 activity, regulation of replication-origin firing, and maintenance of the G2/M checkpoint
Immediate consequences of inhibition	Spindle defects, spindle assembly checkpoint activation, and prolonged mitotic arrest	Increased origin firing and replication stress, G2/M checkpoint abrogation, and premature mitotic entry
Link to homologous recombination	Impaired PLK1-dependent regulation of RAD51 recruitment and HR competence	CDK1 hyperactivation-induced BRCA2-phosphlrylation impairing RAD51 loading
Consequences in *BRCA1/2*-WT/HR-proficient models	Reduced RAD51 foci, persistence of DNA damage, and mitotic catastrophe	Reduced RAD51 foci, increased replication-associated DNA damage, and premature mitosis leading to mitotic catastrophe
Candidate predictive biomarkers	No clinically validated biomarker; HR proficiency and Cyclin E1 elevation are candidate markers supported by preclinical data	Cyclin E1 overexpression (clinically actionable) and functional replication-stress markers (exploratory); *TP53* alteration alone may be insufficient
Major clinical challenges	Hematologic toxicity and limited efficacy in biomarker-unselected populations	Hematologic and gastrointestinal toxicity and reduced tolerability of some combination regimens
Rational combination strategies	DNA-damaging agents and immune-checkpoint inhibitors	Gemcitabine or platinum agents and immune-checkpoint inhibitors

CDK, cyclin-dependent kinase; HR, homologous recombination.

## 3. Mechanistic framework: HR proficiency as a therapeutic liability

### 3.1. Central hypothesis: why HR-proficient tumors may be vulnerable to checkpoint kinase inhibition

A key finding demonstrated in a limited number of experimental systems is that *BRCA1/2*-WT/HR-proficient ovarian cancer cells are substantially more sensitive to PLK1 or WEE1 inhibition than *BRCA1/2*-mutant/HR-deficient cells [20]. In the models examined, HR-proficient cells depended on checkpoint-coupled HR and orderly mitotic progression. WEE1 inhibition promoted premature mitotic entry with unresolved DNA damage, whereas PLK1 inhibition caused spindle defects and mitotic arrest, both of which were associated with reduced HR-associated repair. By contrast, *BRCA1/2*-mutant/HR-deficient cells showed relative resistance, possibly attributable to compensatory DNA-PKcs-dependent NHEJ. However, the relationship between HR status and WEE1 inhibitor sensitivity may vary across experimental systems. In an independent study using HGSOC cells, adavosertib activity was not associated with HR status [33]. Thus, HR proficiency alone may not be a robust biomarker for the response to WEE1 inhibition. Rather, it may represent one component of a broader vulnerability state that includes replication stress, Cyclin E1/*CCNE1* dysregulation, and loss of G1/S checkpoint function, although the relative contributions of these factors remain unclear.

Within this framework, mitotic catastrophe represents a prominent consequence of checkpoint disruption. It is initiated by aberrant mitosis in the presence of unresolved DNA damage or spindle defects, culminating in cell death or irreversible arrest. In addition, depending on the extent and timing of replication stress, DNA damage, and mitotic perturbation, PLK1 or WEE1 inhibition may induce replication catastrophe, apoptosis, senescence, micronuclei formation, and the activation of innate immune signaling [34–36], which may collectively contribute to their antitumor effects.

### 3.2. PLK1 inhibition: spindle assembly defects and impaired HR-associated repair

Volasertib (BI 6727), a selective PLK1 inhibitor, induces prominent spindle assembly defects. In *BRCA1/2*-WT cells, PLK1 inhibition triggers M-phase accumulation and mitotic arrest, consistent with disrupted spindle organization [37–39]. Critically, volasertib markedly reduced RAD51 foci formation in *BRCA1/2*-WT cells, consistent with impaired HR-associated repair [20]. Because RAD51 foci formation is cell-cycle dependent, this finding should ideally be interpreted in light of the proportion of S/G2-phase cells, which was not specifically assessed in our previous study. The reduction may partly reflect the concurrent shift in cell-cycle distribution and PLK1 inhibition-induced mitotic arrest, rather than necessarily indicating direct suppression of HR activity (Section 5.1). Consequently, DNA lesions that would ordinarily be repaired in S/G2 through HR may persist unresolved into defective mitosis, where spindle assembly failures and reduced HR-associated repair may converge to promote mitotic catastrophe (Figure 2).

**Figure 2.**
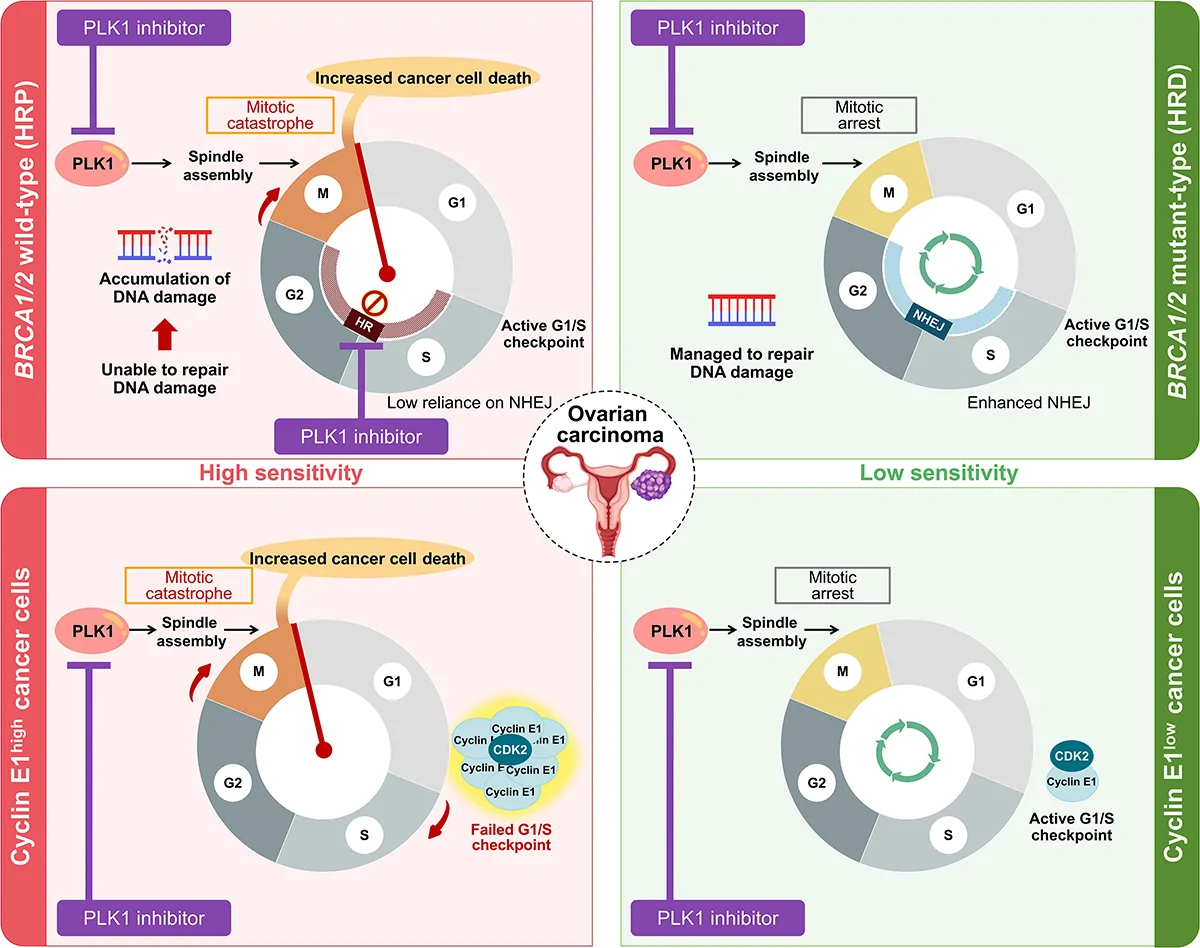
Differential responses to PLK1 inhibition in HR-proficient versus HR-deficient ovarian cancer cells. Upper panels: in *BRCA1/2* wild-type (HR-proficient; HRP) cells (left), PLK1 inhibition disrupts spindle assembly, suppresses homologous recombination, and promotes accumulation of unrepaired DNA damage, resulting in M-phase accumulation, mitotic arrest, and ultimately mitotic catastrophe with increased cancer cell death. In *BRCA1/2* mutant (HR-deficient; HRD) cells (right), PLK1 inhibition likewise disrupts spindle assembly; however, these cells can compensate for DNA damage through enhanced NHEJ, resulting in relative resistance and mitotic arrest without catastrophic cell death. Lower panels: in Cyclin E1-high cancer cells (left), Cyclin E1 overexpression compromises the G1/S checkpoint, and PLK1 inhibition further disrupts spindle assembly, is associated with reduced RAD51 foci and impaired HR-associated repair, and promotes accumulation of unrepaired DNA damage, resulting in prominent M-phase accumulation, mitotic arrest, and mitotic catastrophe. In Cyclin E1-low cancer cells (right), PLK1 inhibition induces less pronounced mitotic disruption and confers lower therapeutic sensitivity. This schematic represents a conceptual model derived primarily from a limited number of experimental studies [20,29]; the relative contributions of HR status and Cyclin E1 elevation to the depicted phenotypes have not yet been independently validated across multiple model systems.

### 3.3. WEE1 inhibition: replication stress amplification and checkpoint abrogation

Adavosertib (MK-1775/AZD1775), a WEE1 inhibitor, acts through a distinct mechanism: it abrogates the G2/M checkpoint, forcing premature mitotic entry before DNA damage is resolved. WEE1 inhibition elevates CDK1 and CDK2 activity, amplifying replication stress and interfering with BRCA2-dependent RAD51 loading [40–42]. In our *BRCA1/2*-WT ovarian cancer models, adavosertib reduced RAD51 foci and increased γH2AX, consistent with impaired HR-associated repair in the setting of increased DNA damage [20]; the same cell cycle caveat noted in Section 3.2 applies to this finding. By simultaneously increasing the DNA damage burden and compromising HR-associated repair, WEE1 inhibition may drive HR-proficient cells toward mitotic catastrophe (Figure 3).

**Figure 3.**
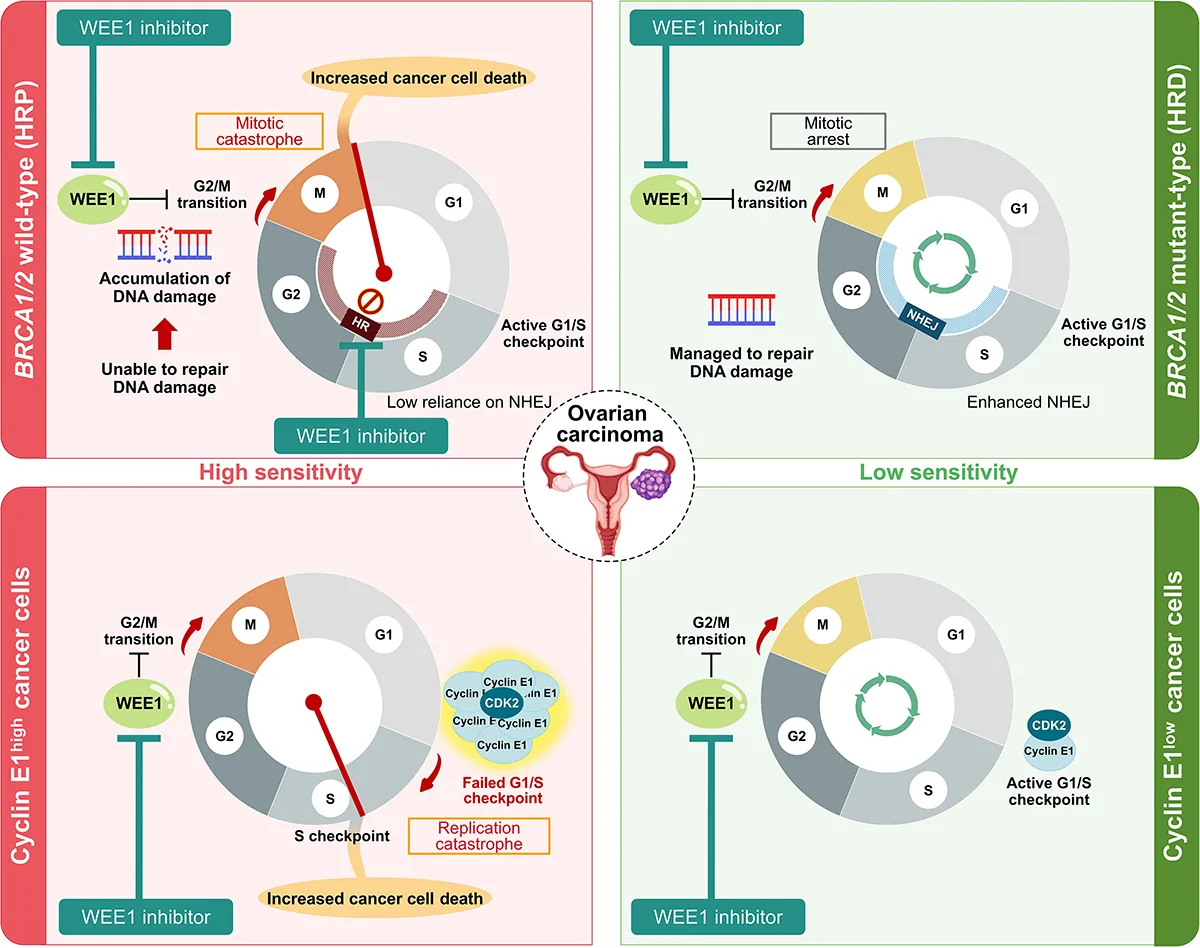
Differential responses to WEE1 inhibition in HR-proficient versus HR-deficient ovarian cancer cells. Upper panels: in *BRCA1/2* wild-type (HR-proficient; HRP) cells (left), WEE1 inhibition abrogates the G2/M checkpoint, leading to premature mitotic entry, reduced RAD51 foci consistent with impaired HR-associated repair, accumulation of unrepaired DNA damage, and mitotic catastrophe, ultimately resulting in increased cancer cell death. In *BRCA1/2* mutant (HR-deficient; HRD) cells (right), WEE1 inhibition likewise perturbs cell-cycle control; however, these cells can compensate for DNA damage through enhanced NHEJ, resulting in relative resistance and mitotic arrest rather than catastrophic cell death. Lower panels: in Cyclin E1-high cancer cells (left), Cyclin E1 overexpression compromises the G1/S checkpoint and enhances CDK2-driven replication stress. WEE1 inhibition further increases unscheduled cell-cycle progression and DNA damage, culminating in mitotic catastrophe and increased cancer cell death. In Cyclin E1-low cancer cells (right), the G1/S checkpoint remains relatively intact, and WEE1 inhibition induces less replication stress and lower therapeutic sensitivity. As with Figure 2, this schematic depicts a conceptual model based primarily on findings from a few studies [20,29], and has not yet been independently validated across additional model systems.

### 3.4. Compensatory NHEJ and DNA-PKcs synthetic lethality

*BRCA1/2*-mutant cells exhibited low sensitivity to either PLK1 or WEE1 inhibition in our models [20], possibly reflecting, at least in part, compensatory DNA-PKcs-dependent NHEJ. Under the experimental conditions, these cells predominantly underwent mitotic arrest rather than cell death. Experimental support for this compensatory mechanism comes from the restoration of *BRCA1/2*-mutant cell sensitivity following small interfering RNA (siRNA) knockdown or pharmacological inhibition of DNA-PKcs (NU7441) [20]. However, the broader proposal that HR-deficient cells preferentially rely on DNA-PKcs-dependent NHEJ following PLK1 or WEE1 inhibition represents a mechanistic interpretation of this synthetic lethality result rather than a directly demonstrated general principle. Whether PLK1-dependent TMEJ contributes to the differential sensitivity of HR-proficient and HR-deficient cells to PLK1 inhibition remains unknown and was not examined in the models discussed here. These findings support a model in which HR-proficient cells rely on checkpoint-coupled HR, whereas HR-deficient cells survive through compensatory DNA-PKcs-dependent NHEJ.

## 4. Clinical development of PLK1 and WEE1 inhibitors: unmet expectations and context-dependent efficacy

### 4.1. Summary of clinical trials

Table 2 summarizes selected clinical trials of PLK1 and WEE1 inhibitors. PLK1 inhibitors have generally shown modest or inconclusive efficacy, whereas WEE1 inhibitors have demonstrated consistent clinical activity in biomarker-selected populations. Cyclin E1 protein overexpression has emerged as a promising predictive biomarker of WEE1 inhibitor sensitivity.

**Table 2.** Selected clinical trials illustrating the clinical development, biomarker strategies, and current status of PLK1 and WEE1 inhibitors.

Agent	Disease / Setting	Phase / Status	Biomarker / Selection	Regimen	Key Clinical Findings	Major Toxicity	Reference / NCT
**PLK1 Inhibitors**							
Volasertib	Platinum-resistant/refractory OC	Phase II	None	Monotherapy	No efficacy benefit vs. chemotherapy; primary endpoint (24-wk DCR) not met: 30.6% vs 43.1%; PFS not improved (13.1 vs 20.6 wk, HR 1.01); 11% of volasertib patients had durable PFS > 1 year	Neutropenia, thrombocytopenia, anemia	Pujade-Lauraine 2016 [43] PMID 26755507 NCT01121406
Volasertib	Acute myeloid leukemia	Phase III	None	LDAC ± volasertib	No clinical benefit; primary endpoint ORR not significantly improved: 25.2% vs 16.8% (OR 1.66, 95% CI 0.95–2.89, *p* = 0.071); key secondary endpoint OS not improved: 5.6 vs 6.5 mo (HR 0.97)	Febrile neutropenia, infections, fatal AEs	Döhner 2021 [44] PMID 34350385 NCT01721876
Onvansertib	2 L *KRAS*-mutant mCRC	Phase II	*KRAS*-mutant	+ FOLFIRI + bevacizumab	Overall ORR 26.4%; bevacizumab-naïve subgroup ORR 76.9% vs 10.0% with prior bevacizumab exposure	Neutropenia	Ahn 2025 [45] PMID 39475591 NCT03829410
Onvansertib	1 L RAS-mutant mCRC (CRDF-004)	Phase II, randomized (interim)	RAS-mutant	30 mg + FOLFIRI + bevacizumab vs FOLFIRI + bevacizumab	Interim data (2026 ASCO): confirmed ORR 72.2% vs 42.1% with control; Phase III registrational trial planned	Neutropenia (G ≥ 3)	Lenz 2026 (ASCO) [46] NCT06106308
**WEE1 Inhibitors**							
Adavosertib	Platinum-resistant OC	Phase II, randomized	None (*CCNE1* exploratory, post hoc)	+ gemcitabine	First RCT to show OS benefit in platinum-resistant/refractory OC: PFS 4.6 vs 3.0 mo (HR 0.55, *p* = 0.015); OS 11.4 vs 7.2 mo (HR 0.56, *p* = 0.017); ORR 23% vs 6%; *CCNE1*-amplified tumors had significantly higher ORR (post hoc, *p* = 0.013)	Neutropenia, thrombocytopenia, fatigue; 1 sepsis-related death	Lheureux 2021 [47] PMID 33485453 NCT02101775
Adavosertib	Uterine serous carcinoma	Phase II	None (*TP53*-altered in 100% tested)	Monotherapy	ORR 29.4%; *TP53* alteration in 100% (32/32 tested); *CCNE1* amplification/gain in 31%.	Diarrhea, anemia, fatigue	Liu 2021 [48] PMID 33705205 NCT03668340
Adavosertib	TP53-mutant platinum-resistant OC	Phase II	*TP53*-mutant	+ carboplatin	Part 1: ORR 43%, PFS 5.3 mo; Part 2 (biomarker-enriched): ORR 41%, PFS 5.6 mo; *CCNE1* amplification trend toward better response.	Neutropenia	Leijen 2016 [49]; Embaby 2023 [50] PMID 27998224 / 37236033 NCT01164995
Adavosertib	Recurrent platinum-resistant HGSOC (IGNITE)	Phase II	Cyclin E1 overexpression (IHC H-score > 50), with or without CCNE1 amplification	Monotherapy; 300 mg QD (D1–5, D8–12 of 21-day cycle); later reduced to 200 mg QD for safety	Cohort 2 (overexpressed, non-amplified): ORR 53%, CBR 61% — substantially higher than historical unselected adavosertib ORR.	Neutropenia, fatigue (dose reduction required in 53% of patients)	Au-Yeung 2022 (ASCO) [51] ACTRN12619001185156
Adavosertib	CCNE1-amplified solid tumors (OC-enriched)	Phase II	*CCNE1*-amplified	Monotherapy	Promising activity concentrated in *CCNE1*-amplified tumors; ORR 27% overall, 36% in the OC cohort.	Gastrointestinal toxicity, anemia, fatigue	Fu 2023 [52] PMID 36469840 NCT03253679
Azenosertib (ZN-c3)	Cyclin E1+ platinum-resistant HGSOC (DENALI)	Phase II (Part 1b completed; Part 2 prospective biomarker-selected, ongoing)	Cyclin E1 IHC-positive (required for Part 2)	Monotherapy; Part 1b 400 mg QD (5-on/2-off); Part 2a dose-confirmation 300 vs 400 mg QD	Part 1b: Cyclin E1+ predictive biomarker confirmed, ORR 32.6% (Cyclin E1 IHC+) / 30.4% (*CCNE1*-amplified). Part 2: ongoing, no efficacy data reported yet.	Fatigue, thrombocytopenia, neutropenia (G ≥ 3); 2 treatment-related G5 AEs previously reported	Simpkins 2025 [Part 1b] Leary 2025 [Part 2] NCT05128825
Azenosertib (ZN-c3)	Cyclin E1+ platinum-resistant HGSOC (ASPENOVA)	Phase III	Cyclin E1 IHC-positive	Monotherapy; 400 mg QD (5-on/2-off) vs investigator’s-choice chemotherapy	Ongoing (target ~420 patients); first patient dosed May 2026; primary endpoint PFS, key secondary OS/ORR; confirmatory trial to convert DENALI accelerated approval to full approval.	Not yet reported	Zentalis 2026 (press release) [53] NCT07546500

Trials were selected to highlight late-stage efficacy or failure, biomarker-enriched clinical signals, and ongoing registration-intent development; early dose-escalation studies without clear translational implications were omitted.

OC denotes ovarian, primary peritoneal, and/or fallopian tube cancer according to the eligibility criteria of the source trial.

1 L, first-line; 2 L, second-line; AE, adverse event; CBR, clinical benefit rate; CI, confidence interval; DCR, disease control rate; FOLFIRI, folinic acid, fluorouracil, and irinotecan; HGSOC, high-grade serous ovarian cancer; HR, hazard ratio; IHC, immunohistochemistry; LDAC, low-dose cytarabine; mCRC, metastatic colorectal cancer; OC, ovarian cancer; OR, odds ratio; ORR, overall response rate; OS, overall survival; PFS, progression-free survival; QD, once daily; RCT, randomized controlled trial.

### 4.2. PLK1 inhibitors: limited clinical success despite strong biological rationale

PLK1 inhibitors have demonstrated strong preclinical activity but limited clinical success, reflecting a disconnect between mechanistic understanding and effective translation (Table 2). Volasertib showed activity in early-phase studies but failed to demonstrate superior progression-free survival compared with chemotherapy in platinum-resistant ovarian cancer, and was associated with significant hematologic toxicity [43,54]. This limited clinical efficacy may reflect several factors, including (1) the absence of biomarker-driven patient selection; (2) a narrow therapeutic window related to on-target toxicity in proliferating normal tissues, particularly hematopoietic cells; and (3) intrinsic or acquired resistance mechanisms. In a pivotal phase III acute myeloid leukemia (AML) trial, volasertib combined with low-dose cytarabine was associated with an increased incidence of fatal adverse events and early mortality, particularly from myelosuppression-related infections, potentially offsetting its clinical benefit [44].

Recently, onvansertib has shown encouraging preclinical and clinical activity. In *KRAS*-mutant metastatic colorectal cancer treated with onvansertib plus FOLFIRI (folinic acid, fluorouracil, and irinotecan) and bevacizumab, the overall response rate was 26.4%; in an exploratory subgroup of bevacizumab-naïve patients, it was 76.9% [45]. Thus, the clinical potential of PLK1 inhibition may depend on disease context, rational combinations, and biomarker-guided patient selection. Biomarker-driven clinical trials of PLK1 inhibitors remain scarce, despite accumulating preclinical evidence supporting molecular stratification (Table 2).

Preclinical studies have identified several potential mechanisms of resistance to PLK1 inhibition, including mutations in the PLK1 ATP-binding domain and increased MDR1 expression in AML cells [55,56] and altered mitochondrial protein expression signatures in melanoma models [57]. None of these mechanisms has yet been validated in ovarian cancer. This contrasts with WEE1 inhibitor development, in which Cyclin E1/*CCNE1*-high enrichment was incorporated into clinical development from a comparatively early stage (Section 4.3), resulting in stronger clinical support for the association between Cyclin E1 and WEE1 inhibitor sensitivity. Clinical programs linking Cyclin E1 to PLK1 inhibitor sensitivity remain limited, and this difference in developmental history may partly account for the current disparity in the evidence levels between the two targets.

### 4.3. WEE1 inhibitors: clinical activity shaped by molecular context

By contrast, WEE1 inhibition has demonstrated modest but reproducible clinical activity (Table 2). Tumors with p53 deficiency or elevated replication stress exhibit heightened dependence on the G2/M checkpoint function [49,58,59]. Cyclin E1 overexpression has emerged as a promising predictive biomarker in ovarian carcinomas [58,60]. A randomized phase II trial demonstrated that adavosertib combined with gemcitabine improved progression-free survival in platinum-resistant ovarian cancer [47], and additional studies have reported its activity in *TP53*-mutant, platinum-resistant settings when combined with carboplatin [49]. This activity has also been observed in *CCNE1*-amplified tumors [52].

However, the determinants of WEE1 inhibitor efficacy remain incompletely understood. Single-agent responses are modest, reflecting a dependence on specific molecular contexts that are inconsistently identified in practice. Although many combination strategies have been developed based on a mechanistic rationale, biomarker-guided patient selection has not yet been systematically incorporated into most clinical trials, and overlapping toxicities may limit the dose intensity. Preclinical studies have identified potential mechanisms of resistance to WEE1 inhibition, including compensatory upregulation of PKMYT1 (Myt1), which maintains inhibitory CDK1 phosphorylation independently of WEE1, and activation of the mechanistic target of rapamycin (mTOR) pathway as an adaptive response to replication stress [61,62]. Recent clinical developments have increasingly adopted biomarker-driven trial designs, particularly those based on Cyclin E1 status. Collectively, these findings support a clinical development strategy that focuses on biologically defined target populations for WEE1 inhibition. The ongoing phase III ASPENOVA trial [53] is evaluating azenosertib monotherapy against investigator’s choice chemotherapy (paclitaxel, pegylated liposomal doxorubicin, gemcitabine, or topotecan) in patients with Cyclin E1-positive, platinum-resistant ovarian cancer (Table 2). By restricting enrollment to this biomarker-defined subgroup, the trial design directly addresses the patient selection limitations discussed above.

## 5. Biomarker development and clinical translation

### 5.1. Limitations of static genomic markers and the need for functional assessment

Clinical experience with PLK1 and WEE1 inhibitors suggests the need for a shift from static genomic stratification to functional biomarkers that capture the current DNA repair capacity and replication stress. Current HR deficiency (HRD)-based stratification in ovarian cancer relies on genomic instability scar assays (loss of heterozygosity, telomeric imbalance, and large-scale state transitions), which predict PARP inhibitor response but reflect historical rather than current HR repair status [63]. HR status is inherently dynamic; *BRCA1/2*-mutant tumors may acquire *BRCA1/2* reversion mutations or loss of 53BP1/Shieldin, restoring HR function and conferring resistance [64,65]. Conversely, replication stress and checkpoint perturbations dynamically modulate HR activity across treatment phases [66]. Checkpoint kinase inhibition itself may transiently impair HR in otherwise HR-proficient tumors, potentially conferring PARP inhibitor sensitivity [67]. These dynamics indicate that HR status is not fixed but evolves, motivating the functional biomarker approaches described below. Accordingly, the discordance between genomic HRD status and functional HR assessment may identify tumors with restored HR activity. A tumor may retain historical genomic HRD scars while showing preserved RAD51 foci formation, which is consistent with current HR proficiency. However, functional assays alone cannot determine the mechanisms of HR restoration, such as *BRCA1/2* reversion, which requires complementary molecular analyses.

Two complementary functional approaches have been developed for this purpose. First, RAD51 foci formation provides a functional readout of current HR competence and can distinguish HR-deficient from HR-proficient tumors. Among the approaches reported to date, it comes closest to capturing this real-time repair capacity, although it has not yet been established as a routine clinical assay [68,69]. Because RAD51 foci are formed primarily in S/G2-phase cells, interpretation should account for proliferation and preferably quantify RAD51 foci within geminin-positive tumor cells. Concurrent assessment of DNA damage, such as γH2AX, can help confirm that reduced RAD51 foci reflect impaired repair rather than insufficient damage induction [68]. Assay thresholds and scoring methods must be standardized [68,69]. Second, replication stress stratification based on genomic and molecular surrogates, including *CCNE1* amplification/overexpression, *RB1* or *MYC* dysregulation, and Cyclin E1/CDK2 activation, has been associated with response to checkpoint kinase inhibition [70]. Functional assessment using phospho-RPA2 (p-RPA2) immunofluorescence in formalin-fixed, paraffin-embedded specimens, which is not yet a routine clinical assay, further stratifies HR-proficient tumors and predicts platinum and PARP inhibitor response [71].

We classified biomarker maturity as exploratory, preclinically validated, or clinically actionable, based on the level of biological, preclinical, and clinical evidence. Within this framework, Cyclin E1 is the most clinically advanced biomarker for WEE1 inhibition and is categorized as clinically actionable in the context of biomarker-guided clinical development, supported by the feasibility of immunohistochemistry (IHC)-based assessment and clinical enrichment strategies (Section 4.3). RAD51 foci and p-RPA2 remain exploratory, pending further validation and assay standardization. For PLK1 inhibition, no clinically established biomarker is currently available; however, HR proficiency and Cyclin E1 elevation remain candidate biomarkers supported by preclinical validation.

Rather than constituting a ready-to-use clinical biomarker panel, the combination of Cyclin E1/*CCNE1* status, RAD51 foci formation, and p-RPA2 expression provides a rational translational framework for patient stratification. Clinical assessment of Cyclin E1 is supported by both the reproducibility of clinical-grade IHC assays (90% inter-pathologist concordance for positivity status) and the high prevalence of Cyclin E1 overexpression (approximately 59%) across HGSOC cohorts, more than half of which lack *CCNE1* amplification [58]. Further prospective studies integrating functional and genomic biomarkers are required before such an integrated approach can be incorporated into clinical decision-making.

### 5.2. Combination strategies

Two rational combination approaches warrant further clinical investigation. First, combining checkpoint kinase inhibitors with conventional DNA-damaging agents may further enhance therapeutic efficacy. Gemcitabine, carboplatin, and topoisomerase inhibitors increase DNA damage and replication stress. The inhibition of either PLK1 or WEE1 may further increase the consequences of DNA damage by amplifying replication stress and/or preventing checkpoint-mediated recovery, thereby enhancing cytotoxicity. Clinical benefits have been demonstrated for adavosertib when combined with gemcitabine [47] and carboplatin [49], although its efficacy remains limited in unselected patient populations. Second, checkpoint kinase inhibitors may be combined with immunotherapy. WEE1 inhibition has been reported to activate innate immune signaling through endogenous retrovirus (ERV)/double-stranded RNA-mediated interferon responses in ovarian cancer models, and preclinical activity has been demonstrated in combination with programmed death-ligand 1 blockade [72]. By contrast, evidence for WEE1-associated cGAS-STING activation and PLK1-induced immunogenic cell death has been derived mainly from non-ovarian preclinical models [73–75]. Clinical validation of checkpoint kinase inhibitor/immunotherapy combinations for ovarian cancer is not yet available.

In addition, because overlapping toxicities may constrain concurrent treatment, sequential or intermittent schedules should also be considered. The differential sensitivity of proliferating tumor and normal cells to checkpoint kinase inhibition may arise, at least in part, from differences in checkpoint dependence. Compared with many *TP53*-altered tumor cells, normal proliferating cells generally retain relatively intact G1/S checkpoint control and may therefore be less dependent on G2/M checkpoint function, providing a partial molecular basis for a therapeutic window. However, because PLK1 and WEE1 also regulate essential mitotic and replication processes in normal proliferating cells, this window is inherently limited, particularly in rapidly renewing tissues, such as the hematopoietic and gastrointestinal compartments [76]. This is clinically reflected in the hematologic and gastrointestinal toxicities observed with PLK1 and WEE1 inhibitors. Prospective studies are required to define the efficacy, toxicity, optimal scheduling, and biomarker-defined tumor context before broader clinical implementation.

## 6. Future perspectives and conclusions

*BRCA1/2*-WT and HR-proficient tumors remain clinically challenging because, unlike *BRCA1/2*-mutant tumors, they retain sufficient DNA repair capacity to withstand PARP inhibitors and other DNA damage-directed therapies. The evidence discussed here suggests that this repair competence may be linked to intact checkpoint regulation and co-occurring features such as replication stress and Cyclin E1 elevation, which together may contribute to therapeutic vulnerability.

PLK1 and WEE1 offer mechanistically distinct approaches within the G2/M checkpoint axis by disrupting checkpoint control and HR-associated repair. PLK1 inhibition induces spindle assembly defects, whereas WEE1 inhibition amplifies replication stress and promotes premature mitotic entry. Despite these distinct upstream mechanisms, both can restrict the compensatory capacity of HR-proficient tumor cells, thereby promoting mitotic catastrophe and cell death. This convergent disruption may contribute to the selective activity observed in reported HR-proficient models.

However, clinical translation requires better integration of the molecular context into the trial design. Functional biomarkers of HR competence and ongoing replication stress (RAD51 foci and p-RPA2), together with molecular surrogates of replication stress susceptibility (Cyclin E1 overexpression), should be prospectively evaluated for patient selection. In addition, rational scheduling and biomarker-guided combinations are important to maximize efficacy and limit toxicity. Future clinical development should preserve the shared framework of G2/M checkpoint vulnerability while accounting for the distinct pharmacological consequences of PLK1 and WEE1 inhibition. As the optimal treatment schedules, combination partners, and dose-limiting toxicities are unlikely to be identical, agent-specific trial designs are required. Biomarker-enriched early phase studies incorporating Cyclin E1/CCNE1 status together with pharmacodynamic assessment of RAD51 foci or replication-stress markers in paired specimens, where feasible, may help identify tumors in which checkpoint inhibition produces the intended functional impairment of HR. Ultimately, targeting the mechanisms sustaining HR proficiency rather than focusing solely on HR deficiency may offer a promising therapeutic avenue for this clinically challenging tumor population.

## Data Availability

No new data were generated or analyzed in support of this review.
